# Exploring the Causality Between Hypothyroidism and Non-alcoholic Fatty Liver: A Mendelian Randomization Study

**DOI:** 10.3389/fcell.2021.643582

**Published:** 2021-03-15

**Authors:** Shizheng Qiu, Peigang Cao, Yu Guo, Haoyu Lu, Yang Hu

**Affiliations:** ^1^School of Life Sciences and Technology, Harbin Institute of Technology, Harbin, China; ^2^Department of Cardiovascular, General Hospital of Heilongjiang Province Land Reclamation Bureau, Harbin, China

**Keywords:** Mendelian randomization, hypothyroidism, NAFLD, causality, GWAS, SNPs

## Abstract

The etiology of non-alcoholic fatty liver disease (NAFLD) involves complex interaction of genetic and environmental factors. A large number of observational studies have shown that hypothyroidism contributes to a high risk of NAFLD. However, the exact causality is still unknown. Due to the progress of genome-wide association study (GWAS) and the discovery of Mendelian randomization (MR), it is possible to explore the causality between the two diseases. In this study, in order to research into the influence of intermediate phenotypes on outcome, nine independent genetic variants of hypothyroidism obtained from the GWAS were used as instrumental variables (IVs) to perform MR analysis on NAFLD. Since there was no heterogeneity between IVs (*P* = 0.70), a fixed-effects model was used. The correlation between hypothyroidism and NAFLD was evaluated by using inverse-variance weighted (IVW) method and weighted median method. Then the sensitivity test was analyzed. The results showed that there was a high OR (1.7578; 95%CI 1.1897–2.5970; *P* = 0.0046) and a low intercept (−0.095; *P* = 0.431). None of the genetic variants drove the overall result (*P* < 0.01). Simply, we proved for the first time that the risk of NAFLD increases significantly on patients with hypothyroidism. Furthermore, we explained possible causes of NAFLD caused by hypothyroidism.

## Introduction

Non-alcoholic fatty liver disease (NAFLD) is the leading cause of chronic liver disease and affects almost a quarter of the world’s population (Fan et al., 2011; Rinella, 2015; Chalasani et al., 2018). NAFLD covers a range of progressive liver diseases, which usually develops into cirrhosis and hepatocellular carcinoma (Byrne et al., 2018; Younossi et al., 2018). Moreover, NAFLD may increase the risk of heart metabolic diseases (e.g., type 2 diabetes, cardiovascular disease, and chronic kidney disease) (Mantovani et al., 2018). For decades, the mainstream view is that the serious disorder of glucose and lipid metabolism and imbalance of energy homeostasis are possible causes of NAFLD (Byrne and Targher, 2015; Non-alcoholic Fatty Liver Disease Study et al., 2015; Mantovani et al., 2018). Thyroid hormone (TH) happens to be one of the key regulator of energy homeostasis, which has a significant impact on glucose and lipid metabolism (Sinha et al., 2018). Hence, the subject whether hypothyroidism is a risk factor of NAFLD has attracted more attention. Chung et al. (2012) conducted a cross-sectional study with a total of 2,324 pairs of subjects with thyroid dysfunction and their controls. The results demonstrated that hypothyroidism was closely associated with NAFLD (OR: 1.38, 95%CI 1.17–1.62). Ludwig et al. (2015) assessed a cross-sectional study of 1,276 subjects and found that the prevalence of hepatic steatosis rises significantly with reductions in the serum thyroxin concentrations. Both of the studies above indicated that hypothyroidism is a risk factor of NAFLD. However, some other evidences have shown no causality between them (Guo et al., 2018) or even a reverse causality (Pagadala et al., 2012; Mantovani et al., 2018).

**FIGURE 1:**
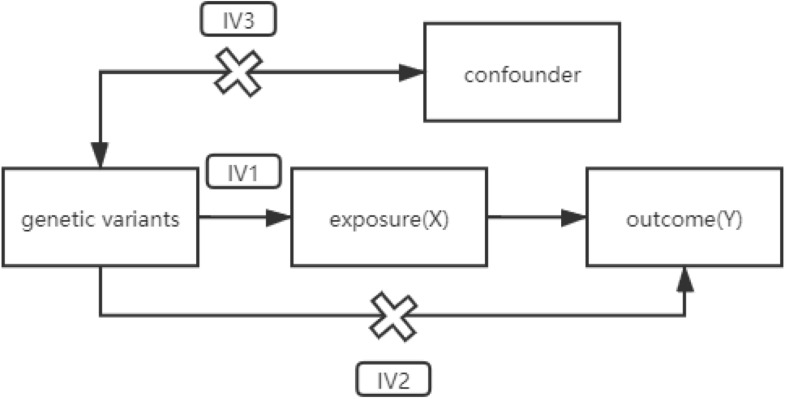
A directed acyclic graph of MR model. IV assumption 1: IVs are strongly correlated with exposure. IV assumption 2: IVs are independent of outcomes (i.e., IVs can only affect outcomes through exposure). IV assumption 3: IVs are not related to confounding factors. MR, Mendelian randomization; IVs, instrumental variables.

Randomized controlled trials (RCTs) are considered to be effective tools to judge the causality in clinical research. Nevertheless, in fact, RCT may be easy to be interfered by confounding factors. In addition, it is difficult to judge the sequence of some exposure and outcome, and the reverse causality is likely to be produced. Consequently, in this study, we designed a Mendelian randomization (MR) experiment to determine the causality between the two diseases. Unlike observational studies, which have many unavoidable limitations, MR estimation uses random allocation of alleles to simulate RCTs. The model based on MR is shown in Figure 1 and needs to satisfy three assumptions (Figure 1; Hemani et al., 2018b).

In our study, genetic variants related to hypothyroidism were introduced as instrumental variables (IVs). The causality between intermediate phenotypes and disease outcomes could be inferred by calculating the Wald ratio of the single-nucleotide polymorphism (SNP) effect on the outcome over the SNP effect on the exposure. According to Mendel’s genetic law, alleles are randomly assigned during separation. The time that each person obtains an allele at this SNP locus must occur before any confounding factors occur. Therefore, analysis based on IVs could exclude the influence of reverse causality.

## Materials and Methods

### Data Source

Two genome-wide association study (GWAS) datasets were used for MR analysis. In order to avoid the potential bias caused by population stratification, we only selected genetic variants and their corresponding aggregate statistics in the study of European origin (Cui et al., 2020). We obtained GWAS data of hypothyroidism from the IEU GWAS database which aggregated and analyzed 10,211 samples and 10,211 controls of European descent, and we obtained the information of 16,152,119 SNPs (Hemani et al., 2018b). The database was developed by the MRC Integrative Epidemiology Unit (IEU) at the University of Bristol. It is a collection of complete GWAS summary datasets, which can be downloaded as open-source files. The NAFLD dataset was obtained from GWAS study published by Anstee et al. (2020), which involved 1,483 European NAFLD cases and 17,781 genetically matched controls. It is the largest histology-based NAFLD GWAS to date, exhibiting the full spectrum of biopsy-proven NAFLD. The study had the necessary ethical approvals from the relevant national/institutional review boards, and all participants provided informed consent.

The corresponding genetic variants, chromosome numbers, positions, effect allele (EA), other alleles, EA frequencies (EAFs), *P*-value, beta coefficients, and standard errors (SEs) were extracted. Among them, the significant hypothyroidism genetic variants (*P* < 5E–08) were extracted to satisfy IV assumption 1, and the insignificant NAFLD genetic variants were extracted to satisfy IV assumption 2. Then their intersection was analyzed by using MR.

### Data Processing and Analysis

Genetic variants should be processed into data that meet IV assumptions. First of all, we removed genetic variants that might have existing linkage disequilibrium (LD). LD refers to the association between alleles on the linked locus (Collins, 2009). They usually tend to be inherited together, which destroy the randomness of genetic variants, and the IV assumptions 2 or 3 may be violated. Thus, the genetic variants with potential LD were removed, and only the parts with longer physical distance (more than 10,000 kb) and less possibility of LD (*R*^2^ < 0.001) were retained. Besides, genetic variants of palindromic and incompatible alleles should be removed when harmonizing exposure and outcome.

### Mendelian Randomization Analysis

The Wald ratio method (Hemani et al., 2018b), inverse-variance weighted (IVW) method (Burgess and Thompson, 2017), and Median-based method (Bowden et al., 2016a) were selected to analyze the relevance between hypothyroidism and NAFLD (Supplementary Methods). Assuming X and Y are the exposure (hypothyroidism) and the outcome (NAFLD), respectively, the Wald ratio (β_*MR*_) of hypothyroidism to NAFLD through specified genetic variants is calculated as follows:

$$\beta_{M\,R}=\beta_{Y}/\beta_{X}$$

and the standard error (σ_*MR*_) of the estimate is

$$\sigma_{M\,R}=\sigma_{Y}/\beta_{X}$$

When there are multiple genetic variants, the IVW estimate (θ_*IVW*_) is the weighted average of these casual estimates, using the inverse of their approximate variances as weights:

θI⁢V⁢W=∑βy⁢βx⁢σy-2∑βx2⁢σy-2

When the intercept is forced to zero, weighted linear regression could be used to estimate the outcome (β_*y*_) genetically related to the exposure (β_*x*_) (Burgess and Thompson, 2017). Fixed-effects IVW assumes that each genetic variant provides the same estimate of the effect; that is, none of the genetic variants shows horizontal pleiotropy. It is mainly applied in cases with no significant heterogeneity. In other cases, the random-effects model should be used (Cheng et al., 2019). Random-effects IVW allows each genetic variant to have a different average effect value (Bowden et al., 2017). It should be noted that the estimates of the random-effects model and the fixed effects model are the same, but the variance of the random-effects model is enlarged in order to take into account the heterogeneity of genetic variants (Hemani et al., 2018b). Furthermore, the weighted median estimate allows more powerful genetic variants to contribute more. It could be obtained by weighting the contribution of each genetic variant according to the inverse-variance associated with the result. Even if up to 50% of the IVs are invalid, the estimator is consistent (Bowden et al., 2016a).

### Heterogeneity Tests

Heterogeneity between IVs is an indicator of potential violations of the IV assumptions (Bowden et al., 2017). Cochran’s Q test needs to be used to calculate the heterogeneity of IVW. Cochran’s Q test follows χ^2^ distribution, and *P* < 0.01 is defined as significant heterogeneity (Cheng et al., 2019).

### Sensitivity Analysis

To evaluate the sensitivity of IVs in MR analysis, we designed a leave-one-out cross-validation (Hemani et al., 2018b). Suppose there are k samples in the genetic variant sets, and we select one genetic variant as the test set and *k* − 1 genetic variants as the training set. It can be evaluated whether the overall results are driven by one genetic variant with a high level of pluripotency. The fluctuation of the results before and after the genetic variant removal reflects the sensitivity of genetic variants. If the estimated value changes greatly when one of the genetic variants is discarded, it can be determined that this genetic variant is an outlier or sensitive.

### Pluripotency Analysis

MR-Egger evaluates whether the pleiotropic effect of genetic variants on the result is different from zero on average (Bowden et al., 2015, 2016b). MR-Egger is similar to IVW, but the former adjusts IVW analysis by allowing non-zero intercepts, namely, allowing horizontal pleiotropic effects. Even if all of the genetic variants violate IV assumption 2, MR-Egger also returns an unbiased estimate of causal effects. In MR-Egger regression, the estimate of intercept can be interpreted as an estimate of the average pleiotropy of all genetic variants, and the slope coefficient provides an estimate of the bias of the causal effect.

The statistical tests for MR analysis were undertaken using the R package of Mendelian Randomization (Yavorska and Burgess, 2017) and TwoSampleMR (Hemani et al., 2018b).

## Results

We calculated the intersection of SNPs, and we kept 13 eligible SNPs after removing LD. Then, we removed the incompatible alleles (rs9272245), and repeated (rs9272245) and palindrome SNPs (rs4766578). The information of nine SNPs that met the requirements, including chromosome numbers, position, beta coefficients, SEs, and *P*-value was shown (Table 1).

**TABLE 1:** Associations of genetic variants between hypothyroidism and NAFLD.

SNP	CHR	Pos	beta.exposure	se.exposure	pval.exposure	beta.outcome	se.outcome	pval.outcome
rs17020127	1	107,815,461	–0.2289	0.0306	7.95E-14	–0.2438	0.1347	0.070361
rs181871363	1	113,318,163	–0.276	0.0456	1.37E-09	–0.2917	0.2025	0.1499
rs11571293	2	203,852,990	0.1259	0.0215	5.11E-09	0.0757	0.0928	0.4147
rs9860547	3	188,411,191	–0.1121	0.0205	4.26E-08	–0.0386	0.0888	0.663599
rs1993945	5	77,222,370	0.1468	0.0201	2.72E-13	0.0558	0.0876	0.5244
rs9272245	6	32,635,095	0.1458	0.025	5.75E-09	0.2047	0.0927	0.7014
rs9273400	6	32,659,351	–0.2252	0.022	1.41E-24	–0.1314	0.095	0.1667
rs925489	9	97,784,318	–0.1887	0.0211	3.31E-19	–0.0102	0.0904	0.9098
rs229536	22	37,193,829	–0.1172	0.0202	6.70E-09	0.0347	0.0879	0.6933

**NAFLD, non-alcoholic fatty liver disease; SNP, single-nucleotide polymorphism.**

### The Influence of Hypothyroidism on the Risk of Non-alcoholic Fatty Liver Disease

The IVW analysis found a significant OR (1.7578; 95%CI 1.1897–2.5970; *P* = 0.0046), proving that hypothyroidism was considerably related to the high risk of NAFLD. Since there was no heterogeneity between genetic variants (*P* = 0.70), a fixed model was preferred for MR analysis. The difference between the results of median-based estimator and the IVW method was very small (OR: 1.7049; 95%CI 1.0021–2.9006; *P* = 0.049). In addition, five other methods were analyzed, including simple median, penalized weighted median, penalized IVW, robust IVW, and penalized robust IVW methods (Table 2; Yavorska and Burgess, 2017). Similar results proved the accuracy of MR analysis.

**TABLE 2:** Five other MR methods to analyze the causality.

Method	Estimate (β)	OR	*P*-value
Simple median	0.583	1.7914	0.038
Penalized weighted median	0.548	1.7298	0.040
Penalized IVW	0.564	1.7577	0.005
Robust IVW	0.562	1.7542	0.001
Penalized robust IVW	0.562	1.7542	0.001

**MR, Mendelian randomization; IVW, inverse-variance weighted.**

### Sensitivity Analysis

**FIGURE 2:**
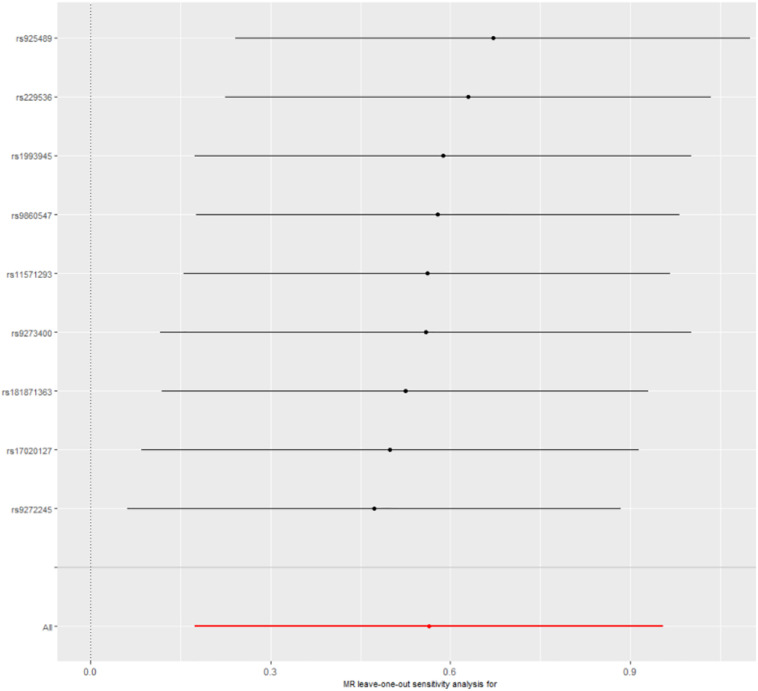
Leave-one-out cross-validation. The red line is the average of all β values after leave-one-out.

Leave-one-out cross-validation was used to calculate the MR result of the remaining IVs after removing them one by one. No matter which genetic variants were removed, the β value was greater than zero; that is, each point had a positive effect on the result (Figure 2 and Supplementary Table 1). There was also no significant difference from the overall result (*P* < 0.01). All the results indicated that none of genetic variants was sensitive.

### Pluripotency Analysis

**FIGURE 3:**
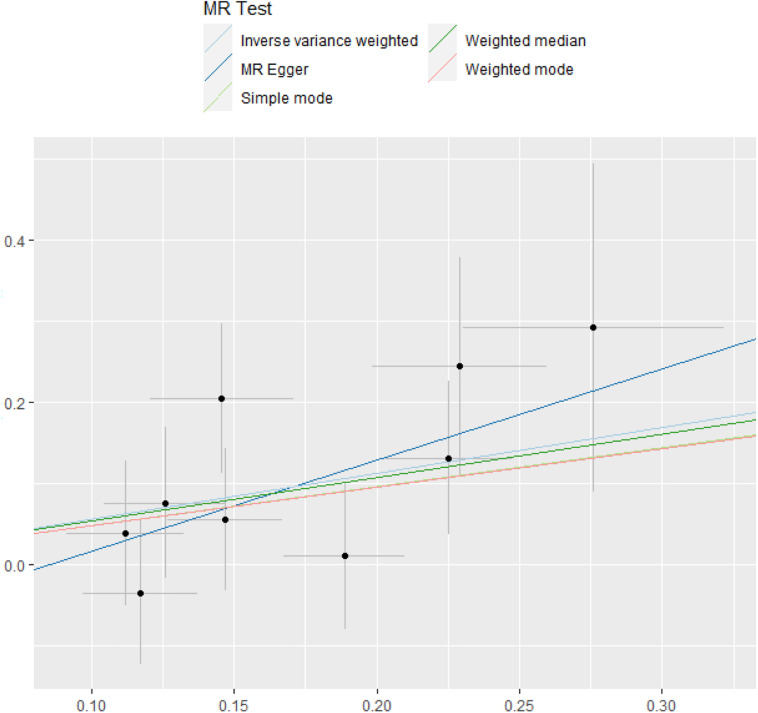
MR test. The estimate of intercept can be interpreted as an estimate of the average pleiotropy of all SNPs, and the slope coefficient provides an estimate of the bias of the causal effect. MR, Mendelian randomization; SNP, single-nucleotide polymorphism.

Five methods were used to evaluate the results of MR analysis, and the scatter plot was generated (Figure 3). Among them, MR-Egger was used to measure the pleiotropy of IVs. There was no statistical difference between the intercept of MR-Egger and the zero intercept of IVW (*P* > 0.05). Moreover, the symmetry of the funnel plot might be another evidence for the absence of horizontal pleiotropy (Supplementary Figure 1; Sterne et al., 2011).

## Discussion

In this study, we used two-sample MR to evaluate the association between hypothyroidism and NAFLD. The high OR (1.7578; 95%CI 1.1897–2.5970; *P* = 0.0046) obtained by IVW method provided a strong evidence that hypothyroidism was associated with an increased risk of NAFLD. Therefore, we speculate that hypothyroidism may induce NAFLD through some metabolic pathway, although the related pathogenesis is still unclear.

In previous studies, the association between hypothyroidism and NAFLD has been researched from various perspectives, both clinically (Chung et al., 2012; Pagadala et al., 2012; Lee et al., 2015; Ludwig et al., 2015; Mantovani et al., 2018; Hu et al., 2019, 2020, 2021) and biologically (Ferrandino et al., 2017; Gionfra et al., 2019; Lonardo et al., 2019). The causality, however, is still unexplored (Efstathiadou et al., 2018). Unlike alcoholic fatty liver disease, NAFLD is often caused by disorder of glucose and lipid metabolism. Hypothyroidism patients have inadequate TH secretion, which is precisely related to the secretion of insulin in adipose tissue and the stimulation of lipolysis by adrenal gland (Pagadala et al., 2012; Ferrandino et al., 2017). If the TH levels in serum are slightly reduced, insulin secretion will be impaired, resulting in reduced lipolysis inhibition (Mullur et al., 2014). Fatty acid traveling to the liver is increased, which induces NAFLD. Interestingly, extremely low TH level may inhibit the development of NAFLD (Ferrandino et al., 2017). Although the pathogenesis of NAFLD is complex, we can still prevent it by avoiding high-sugar and high-fat diet and toning up with more exercise.

Our study benefits from both the GWAS data and MR method. It is noteworthy that the existence of horizontal pleiotropy is a key factor in rejecting the IV assumptions of the MR model (Hemani et al., 2018a). Horizontal pleiotropy means that genetic variants have multiple independent ways to affect outcome (Davies et al., 2018; Hemani et al., 2018a; Wootton et al., 2018). In our study, the low intercept and high *P*-value (-0.095; *P* = 0.431) of MR-Egger seemed to demonstrate that there was no horizontal pleiotropy affecting the results of MR analysis. But we found that the funnel plot generated by MR-Egger was not symmetrical enough. Horizontal pleiotropy might exist because of factors beyond our control, such as population stratification (Persyn et al., 2018) and canalization (Geiler-Samerotte et al., 2019; Hallgrimsson et al., 2019). Meanwhile, due to the lack of relevant data, confounders associated with NAFLD were not explored. Other genetic variants that might be significantly associated with NAFLD should have removed, such as endocrine and metabolic factors (diabetes, hyperlipidemia, etc.).

To the best of our knowledge, our study is the first to reveal the causality between hypothyroidism and NAFLD, which improves the pathogenic factors and patterns of NAFLD. Besides, it helps researchers to understand the relationship between various diseases better from the perspective of system biology (Vidal et al., 2011; Cheng and Hu, 2018; Starchenko and Lauffenburger, 2018).

## Data Availability Statement

The original contributions presented in the study are included in the article/Supplementary Material, further inquiries can be directed to the corresponding author/s.

## Author Contributions

SQ wrote the manuscript and did the experiments. PC provided ideas of this work. YG and HL revised this manuscript and guided how to do experiments. YH supervised this work. All authors contributed to the article and approved the submitted version.

## Conflict of Interest

The authors declare that the research was conducted in the absence of any commercial or financial relationships that could be construed as a potential conflict of interest.
