# Corrigendum: Exploring the Causality Between Hypothyroidism and Non-alcoholic Fatty Liver: A Mendelian Randomization Study

**DOI:** 10.3389/fcell.2021.710566

**Published:** 2021-06-15

**Authors:** Shizheng Qiu, Peigang Cao, Yu Guo, Haoyu Lu, Yang Hu

**Affiliations:** ^1^School of Life Sciences and Technology, Harbin Institute of Technology, Harbin, China; ^2^Department of Cardiovascular, General Hospital of Heilongjiang Province Land Reclamation Bureau, Harbin, China

**Keywords:** mendelian randomization, hypothyroidism, NAFLD, causality, GWAS, SNPs

In the original article, we neglected to include the funder “the National Key R&D Program of China (2017YFC1201201, 2018YFC0910504 and 2017YFC0907503)”, “Heilongjiang Postdoctoral Science Foundation (LBH-Z6064)” and all funding was to “YH”. We also incorrectly stated funding from “the Youth program of National Natural Science Foundation of China (Grant No. 61801147)” which should be “the Natural Science Foundation of China (61801147 and 82003553)”.

The corrected Funding statement appears below:

This work was supported by the National Key R&D Program of China (2017YFC1201201, 2018YFC0910504 and 2017YFC0907503), Heilongjiang Postdoctoral Science Foundation (LBH-Z6064) and the Natural Science Foundation of China (61801147 and 82003553), all to YH.

The authors apologize for this error and state that this does not change the scientific conclusions of the article in any way. The original article has been updated.

