# Supplementary material for: Exploring the Causality Between Hypothyroidism and Non-alcoholic Fatty Liver: A Mendelian Randomization Study
Source: Front Cell Dev Biol. 2021 Mar 15;9:643582. doi: 10.3389/fcell.2021.643582 (PMC8005565; doi:10.3389/fcell.2021.643582)
Supplement: Supplementary file 1 [file Image_1.pdf]

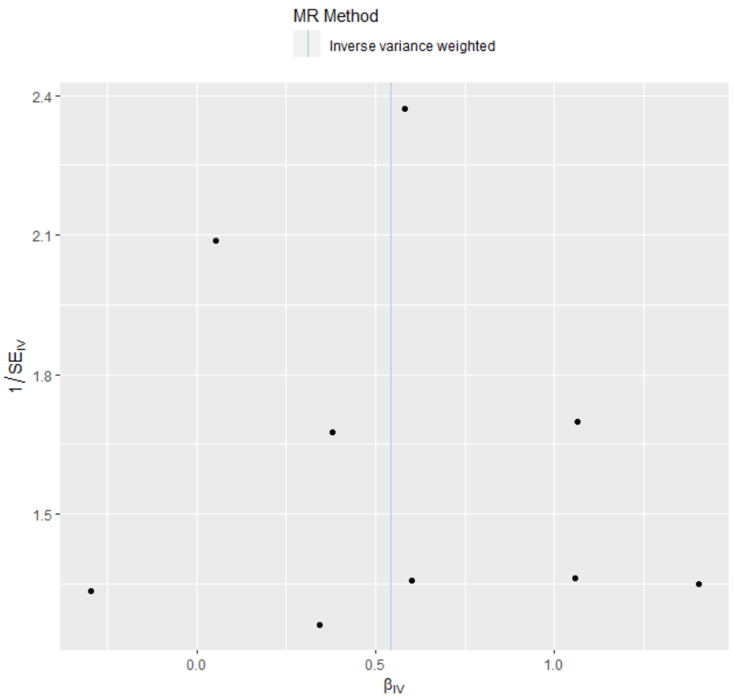


**Supplementary Figure 1 |** The funnel plot. The symmetry of the funnel plot explains the absence of horizontal pleiotropy.
