# Supplementary material for: Exploring the Causality Between Hypothyroidism and Non-alcoholic Fatty Liver: A Mendelian Randomization Study
Source: Front Cell Dev Biol. 2021 Mar 15;9:643582. doi: 10.3389/fcell.2021.643582 (PMC8005565; doi:10.3389/fcell.2021.643582)
Supplement: Supplementary file 2 [file Table_1.docx]

**Supplementary Table 1 |** Leave-one-out cross validation.

| **SNP** | **b** | **OR** | **p** |
| --- | --- | --- | --- |
| rs11571293 | 0.5611152 | 1.752626 | 0.00666996 |
| rs17020127 | 0.4992455 | 1.647478 | 0.018320504 |
| rs181871363 | 0.5248513 | 1.690208 | 0.011192465 |
| rs1993945 | 0.5870987 | 1.798762 | 0.005450359 |
| rs229536 | 0.629286 | 1.876271 | 0.002314717 |
| rs925489 | 0.6705784 | 1.955368 | 0.002194014 |
| rs9272245 | 0.4726842 | 1.604295 | 0.024185028 |
| rs9273400 | 0.5584732 | 1.748002 | 0.013426433 |
| rs9860547 | 0.5788685 | 1.784019 | 0.004901035 |
| All | 0.5640463 | 1.757771 | 0.004620151 |
