# Supplementary material for: Exploring the Causality Between Hypothyroidism and Non-alcoholic Fatty Liver: A Mendelian Randomization Study
Source: Front Cell Dev Biol. 2021 Mar 15;9:643582. doi: 10.3389/fcell.2021.643582 (PMC8005565; doi:10.3389/fcell.2021.643582)
Supplement: Supplementary file 3 [file Table_2.docx]

**Supplementary methods**

**Basic concepts of Mendelian Randomization**

Suppose that J independent genetic variants *G_j_* (*j*=1, 2, ..., *J*) are selected as potential instrumental variables. For each given genetic variant *G_j_* (*j* =1, 2, ..., *J*), the beta coefficients ($\hat{\beta}_{X_{j}}$,$\hat{\beta}_{Y_{j}}$) and their standard errors (*se*($\hat{\beta}_{X_{j}}$), *se*($\hat{\beta}_{Y_{j}}$)) of this genetic variant in exposure X (such as hypothyroidism) and outcome Y (such as NAFLD) are obtained from the GWAS data.

The influence of each genetic variants *G_j_* (*j*=1, 2, ..., *J*) on the outcome Y can be divided into two parts, namely pleiotropic influence and causal influence:

$$\beta_{Y_{j}}=\alpha_{j}+\theta\beta_{X_{j}}$$

Among them, $\alpha_{j}$ represents the influence of the genetic variant *G_j_* (*j*=1, 2, ..., *J*) on outcome Y without exposure X, and $\theta\beta_{X_{j}}$ represents the effect of the genetic variant *G_j_* (*j*=1, 2, ..., *J*) on outcome Y through exposure X. *θ* represents the impact of exposure X on outcome Y. Except through the path of exposure X, if a genetic variant *G_j_* (*j*=1, 2, ..., *J*) is related to multiple causal paths that cause the outcome Y, the genetic variant is called a pleiotropic genetic variant. If $\alpha_{j}$≠0, it means that $\alpha_{j}$ itself contains this pleiotropic effect. Then genetic variant *G_j_* (*j*=1, 2, ..., *J*) violates the assumption of Mendelian randomization, and such genetic variation cannot be used as an input variable. For a given genetic variant *G_j_* (*j*=1, 2, ..., *J*), if $\alpha_{j}$=0, then this variant satisfies the Mendelian randomization assumption and can therefore be used as an input variable (i.e. IVs). For the genetic variant *G_j_* (*j*=1, 2, ..., *J*) that satisfies the Mendelian randomization assumption, the effect of exposure X on outcome Y can be obtained:

$$\hat{\theta_{j}}=\frac{\hat{\beta}_{Y_{j}}}{\hat{\beta}_{X_{j}}}$$

And their approximate variances:

$$v_{j}=\frac{{se(\hat{\beta}_{Y_{j}})}^{2}}{{\hat{\beta}_{X_{j}}}^{2}}$$

**Inverse-variance** **weighted method**

The inverse-variance weighted method (IVW) is a common Mendelian randomization method. For a given single genetic variant *G_j_* (*j*=1, 2, ..., *J*), the influence of exposure X on the outcome Y $\hat{\theta_{j}}=\frac{\hat{\beta}_{Y_{j}}}{\hat{\beta}_{X_{j}}}$ and its variance

$$v_{j}=\frac{{se(\hat{\beta}_{Y_{j}})}^{2}}{{\hat{\beta}_{X_{j}}}^{2}}$$

can be calculated. For multiple independent genetic variants, the IVW method is used for integration to evaluate the impact of the overall exposure X on the outcome Y, and the reciprocal of the variance is used as the weight, namely

$$w_{j}=\frac{1}{v_{j}}$$

The weighted summation of the influence of exposure X of each genetic variant on the result Y, namely

$$\theta_{IVW}=\frac{\sum_{j} \hat{\theta_{j}}*w_{j}}{\sum_{j} w_{j}}$$

$$\theta_{IVW}=\frac{\sum_{j} \hat{\beta}_{Y_{j}}\hat{\beta}_{X_{j}}{se(\hat{\beta}_{Y_{j}})}^{-2}}{\sum_{j} {{\hat{\beta}_{X_{j}}}^{2}se(\hat{\beta}_{Y_{j}})}^{-2}}$$

Furthermore, the above estimated value can also be obtained by a weighted linear regression method, i.e., a weighted linear regression equation of the influence of exposure X on outcome Y is constructed. If the intercept of the regression equation (intercept) is equal to zero, then the regression equation can be expressed as:

$$\hat{\beta}_{Y_{j}}=\theta_{IVW}\hat{\beta}_{X_{j}}+\varepsilon_{{IVW}_{j}}$$

Where $\theta_{IVW}$ represents the effect value (IVW estimate), and the weight is $w_{j}$, which is the inverse variance of $\hat{\beta}_{Y_{j}}$:

$$w_{j}={se(\hat{\beta}_{Y_{j}})}^{-2}$$

Among them, $\varepsilon_{{IVW}_{j}}$is the residual term. If the residual standard error is equal to zero, then the above weighted linear regression method is equivalent to a fixed-effect meta-analysis. If for any genetic variant *G_j_* (*j*=1, 2, ..., *J*), its multi-effect influence is equal to zero, namely $\alpha_{j}$=0 (*j*=1, 2, ..., *J*). In other words, all genetic variants *G_j_* included in the analysis are all valid input variables, then each estimated $\hat{\theta_{j}}$ is consistent with the true causal influence estimate. Therefore, the integrated $\theta_{IVW}$ represents the impact of the overall exposure X on the outcome Y.

**MR-Egger**

The calculation formula of the MR-Egger method is similar to the IVW method, but the MR-Egger can allow the intercept term to be non-zero, namely

$$\hat{\beta}_{Y_{j}}=\theta_{I}+\theta_{E}\hat{\beta}_{X_{j}}+\varepsilon_{E_{j}}$$

Where $\theta_{I}$ represents the intercept term, $\theta_{E}$ represents the MR-Egger estimate, and$\varepsilon_{E_{j}}$ represents the residual term. If the genetic variant *G_j_* (*j*=1, 2, ..., *J*) as an input variable does not have pleiotropy, i.e., $\alpha_{j}$=0, then as the sample size increases, the intercept term gradually becomes zero. Therefore, the MR-Egger estimate ($\theta_{E}$) and IVW $(\theta_{IVW})$ are both consistent with the estimated value of true causal association. If the genetic variant *G_j_* (*j*=1, 2, ..., *J*) as an input variable is pleiotropic, i.e., $\alpha_{j}$≠0, and $\alpha_{j}$ is independently distributed in $\beta_{X_{j}}$, as the sample size and the number of genetic variants increase, the estimated value of MR-Egger ($\theta_{E}$) and the estimated value of true causal association gradually agree.

**Median-based estimator**

The main idea of the weighted median method is based on the robustness of the median relative to the outlying value. Among them, the estimated value of the simple median method can be realized by directly taking the median of the estimated value $\hat{\theta_{j}}=\frac{\hat{\beta}_{Y_{j}}}{\hat{\beta}_{X_{j}}}$ (*j*=1, 2, ..., *J*), and each estimated value has the same weight. The weighted median method takes into account the difference of each estimated value and weights each estimated value. Specifically, for each given estimated value $\hat{\theta_{j}}=\frac{\hat{\beta}_{Y_{j}}}{\hat{\beta}_{X_{j}}}$ (*j*=1, 2, ..., *J*), it is first sorted by its size. Assuming $\hat{\theta_{1}}<\hat{\theta_{2}}<\hat{\theta_{3}}<\ldots<\hat{\theta_{j}}$, where the variance of each estimate is

$$v_{j}=\frac{{se(\hat{\beta}_{Y_{j}})}^{2}}{{\hat{\beta}_{X_{j}}}^{2}}$$

For $\hat{\theta_{j}}$, its weight is defined as $w_{j}$ and $w_{j}$ is its inverse variance weight:

$$w_{j}=\frac{\frac{{\hat{\beta}_{X_{j}}}^{2}}{{se(\hat{\beta}_{Y_{j}})}^{2}}}{\sum_{j} \frac{{\hat{\beta}_{X_{j}}}^{2}}{{se(\hat{\beta}_{Y_{j}})}^{2}}}$$

And the sum of all $w_{j}$ is 1. If it is defined that $S_{k}=\sum_{j\leq k} w_{j}$, it represents the sum of the weights from the 1 to k. If Selecting the largest k that satisfies $s_{k}=\sum_{j\leq k} w_{j}<0.5$, then the weighted median estimate is the weighted average of the k and (k+1) th $\hat{\theta_{j}}$, namely

$$\hat{\theta_{WM}}=\hat{\theta_{k}}+(\hat{\theta_{k+1}}-\hat{\theta_{k}})\times\frac{0.5-s_{k}}{s_{k+1}-s_{k}}$$

If the weights of all estimated values $\hat{\theta_{j}}=\frac{\hat{\beta}_{Y_{j}}}{\hat{\beta}_{X_{j}}}$ (*j*=1, 2, ..., *J*) are equal, the weighted median method is equal to the simple median method.

**Reference**

[1]Burgess, S., and Thompson, S.G. (2017). Interpreting findings from Mendelian randomization using the MR-Egger method. *Eur J Epidemiol* 32**,** 377-389.

[2]Bowden, J., Davey Smith, G., Haycock, P.C., and Burgess, S. (2016a). Consistent Estimation in Mendelian Randomization with Some Invalid Instruments Using a Weighted Median Estimator. *Genet Epidemiol* 40**,** 304-314.

[3]Liu, G., Zhao, Y., Jin, S., Hu, Y., Wang, T., Tian, R., Han, Z., Xu, D., and Jiang, Q. (2018). Circulating vitamin E levels and Alzheimer's disease: a Mendelian randomization study. *Neurobiol Aging* 72**,** 189 e181-189 e189.
